# Pre-clinical investigation of the synergy effect of interleukin-12 gene-electro-transfer during partially irreversible electropermeabilization against melanoma

**DOI:** 10.1186/s40425-019-0638-5

**Published:** 2019-06-26

**Authors:** Lise Pasquet, Elisabeth Bellard, Sophie Chabot, Bostjan Markelc, Marie-Pierre Rols, Justin Teissie, Muriel Golzio

**Affiliations:** Institut de Pharmacologie et de Biologie Structurale, Université de Toulouse, CNRS, UPS, BP 64182, UMR 5089, 205 Route de Narbonne, F-31077 Toulouse Cedex, France

**Keywords:** Gene electrotransfer, Melanoma, Electroporation, IL-12, Immunotherapy

## Abstract

**Background:**

Melanoma is a very aggressive skin tumor that can be cured when diagnosed and treated in its early stages. However, at the time of identification, the tumor is frequently in a metastatic stage. Intensive research is currently ongoing to improve the efficacy of the immune system in eliminating cancer cells. One approach is to boost the activation of cytotoxic T cells by IL-12 cytokine that plays a central role in the activation of the immune system. In parallel, physical methods such as electropermeabilization-based treatments are currently under investigation and show promising results.

**Methods:**

In this study, we set electrical parameters to induce a partial-irreversible electropermeabilization (pIRE) of melanoma to induce a sufficient cell death and potential release of tumor antigens able to activate immune cells. This protocol mimics the situation where irreversible electropermeabilization is not fully completed. Then, a peritumoral plasmid IL-12 electrotransfer was combined with pIRE treatment. Evaluation of the tumor growth and survival was performed in mouse strains having a different immunological background (C57Bl/6 (WT), nude and C57Bl6 (TLR9−/−)).

**Results:**

pIRE treatment induced apoptotic cell death and a temporary tumor growth delay in all mouse strains. In C57Bl/6 mice, we showed that peritumoral plasmid IL-12 electrotransfer combined with tumor pIRE treatment induced tumor regression correlating with a local secretion of IL-12 and IFN-γ. This combined treatment induced a growth delay of distant tumors and prevented the emergence of a second tumor in 50% of immunocompetent mice.

**Conclusions:**

The combination of pIL-12 GET and pIRE not only enhanced survival but could bring a curative effect in wild type mice. This two-step treatment, named Immune-Gene Electro-Therapy (IGET), led to a systemic activation of the adaptive immune system and the development of an anti-tumor immune memory.

**Electronic supplementary material:**

The online version of this article (10.1186/s40425-019-0638-5) contains supplementary material, which is available to authorized users.

## Background

Early diagnosis and treatment of melanoma is essential to cure this aggressive skin tumor. However, the tumor is frequently already metastatic when identified. In this case, even though several treatments are available, the probability for a complete remission are dramatically decreased. Intensive research is currently ongoing to improve the efficacy of the immune system in eliminating cancer cells. One approach consists in blocking the negative regulation of the immune system with checkpoint inhibitor antibodies that block PD1/PDL-1 and/or CTLA-4 immune checkpoints [1]. An alternative approach is boosting the activation of cytotoxic T cells (CTL).

The cytokine interleukin 12 (IL-12) plays a central role in the activation of the immune system [2]. Produced by dendritic cells (DC), macrophages and neutrophils, IL-12 induces the production of interferon γ (INFγ) by activated T cells and NK cells. Moreover, IL-12 induces a positive feedback on its own secretion. Indeed, IL-12-induced IFNγ secretion by CTL and type I helper CD4 T cells (Th1) acts back on DC’s to reinforce their secretion of IL-12 [2]. Therefore, IL-12 has appeared as a key molecule to enhance the anti-tumor immune response. However, as the sustained positive feedback loop induced by recombinant IL-12 administration could lead to high toxicity [3], it is crucial, in the context of IL-12 based therapy, to control the availability of this cytokine in vivo*.* This can be achieved by gene therapy, which consists in the transfer of DNA coding for IL-12 into appropriate target cells. Use of viral vectors, such as lentivirus, lead to a high transduction rate, but display random integration capacities thus hampering their usability for in vivo transfection. Among non-viral methods, Gene-Electro-Therapy (GET) is a promising physical method that brings a controlled level of transfection [4–6]. In vitro, the application of well-defined electric pulses to target cells promotes the entrance of the negatively charged plasmid DNA into the cell resulting in transgene expression [7]. A phase I clinical trial showed that intratumoral GET of plasmid DNA encoding IL-12 (IL-12 GET) resulted in complete regression of melanoma tumors in 2 patients and in a stable disease in 8 patients out of 19 [8]. This was associated with tumor cell necrosis and intratumoral secretion of IL-12. It has been reported that intratumoral GET induces the transfection of only 5% of targeted cells resulting in low levels of transgene expression [5], whereas peritumoral GET induces a better yield of transgene expression [9, 10]. Peritumoral GET, however, loses the advantage of a colocalization with tumor antigen release by lysed tumor cells upon application of electric field. Therefore, it appears that an efficient anti-tumor therapy based on cell electro-permeabilization (EP) should combine a high level of transgene expression with a maximum release of hidden tumor antigens while maintaining low side effects. Several studies using combination of electrochemotherapy and IL-12 gene electrotransfer demonstrated a high antitumor efficacy, also preventing recurrence of distant metastases [11, 12]. Numerous studies suggest involvement of innate and adaptive anti-tumor immune responses induced by IL-12 gene expression and the delivery of the chemotherapeutic agent [10, 13–15].

One method to achieve the release of hidden tumor antigens may be through a strong EP intensity that leads to cell death. This irreversible permeability induced by Electropermeabilization (IRE), is due to the inability of targeted cells to repair the bio-electro-chemical defects of the plasma membrane or the chemical imbalances that occur due to influx and efflux of molecules through these transient or stable defects [16]. These events occur in targeted cells between paired electrodes applied to tissue [17]. This technique has been extensively used for non-thermal ablation of tumors [18] and introduced as cancer treatment in the human clinic [19]. Mechanisms of IRE-induced-cell-death have been widely investigated looking at the cell morphology by histology as well as transmission and scanning electron microscopy [20–22]. The exact pathways required in IRE induced cell death remain under discussion, but it seems that several mechanisms are implicated according to pulsed electric field exposure [20, 23, 24]. In vivo, the effect of IRE has been observed at the tissue-level such as edema (induced by capillary-disruption), hypoxia (induced by temporary vascular occlusion) [25, 26] or possibly, the activation of immune system [27–29]. Importantly, the effect of IRE treatment was shown to be very localized. Tumor tissues treated with IRE exhibited a peak of apoptotic cell death at 24 h after IRE, whereas tissue around the treated area was not affected [30]. In liver and pancreas, IRE treatment was shown to have an anti-tumor effect while still preserving the integrity and function of bile conducts and blood vessels, inducing a slight and transient effect on concentrations of liver and pancreas enzymes [26, 31]. This ability of IRE to achieve cell death immediately adjacent to large vessels without affecting the vessels or peri-ablative tissues made IRE a promising and safe targeted treatment for cancer without drug usage. However, care must be taken in IRE protocols, by carefully choosing pulsed electric field (PEF) parameters to achieve a predominant cell death due to electropermeabilization. Too many pulses and/or a too high tissue conductivity are reported to lead to thermal damages in the immediate vicinity of the electrodes [32]. Furthermore, thermal effects induced by clinical IRE protocols using high energy regimens are reported to moderate the effects of IRE non-thermal ablation [33]. Boosting the IRE induced immune activation was also proposed [27–29].

In this study, we first set PEF parameters to induce a partial-irreversible electropermeabilization (pIRE) of melanoma without using cytotoxic agents. This setting led to cell death and potential release of tumor antigens able of activating the immune system. Our protocol mimicked the situation where irreversible electropermeabilization did not bring a complete eradication of the tumor. The set PEF parameters only induced in vivo partial cell death in the tumor, leading to a delay in tumor growth compared to untreated mice. In addition, we also studied the kinetics of apoptotic tumor cell death induced by PEF treatment. No thermal side effects were observed as demonstrated by the low activation of the thermo-inducible heat-shock protein (Hsp70) promoter 1B (Hspa1b), in double transgenic Hspa1b-LucF (+/+) Hspa1b-mPlum (+/+) mice. Our pIRE treatment was then combined with peri-tumoral IL-12 GET pre-treatment that was shown to induce a local and rapid production of IL-12 [10, 34] and resulted in a rapid production of IFNγ. This treatment induced (i) an increased survival rate of treated animals, (ii) a systemic activation of the adaptive immune system leading to a growth delay of distant tumors and (iii) the development of immune memory that protected against the emergence of a second tumor. We could also discriminate the effect on the tumor growth between the application of peri-tumoral IL-12 GET, pIRE, and the combination of both treatments.

## Methods

### Cell lines

B16F10 mouse melanoma (ATCC, Manassas, VA) and B16F10 cells expressing the enhanced Green Fluorescent Protein (B16F10-eGFP) [35], were grown as monolayer cultures on T75 flasks (Nunc, Denmark) at 37 °C, 5% CO_2_ atmosphere in a humidified chamber until they reached 70% confluence. Culture medium used was Dulbecco’s Modified Eagle Medium with 4.5 g/l D-Glucose and L-Glutamine (DMEM; Gibco/ Life Technologies) supplemented with 10% fetal bovine serum (Sigma-Aldrich, St Louis, MO) and the antibiotics penicillin (100 U/ml) and streptomycin (100 U/ml) (Gibco/ Life Technologies).

### Mice

Female C57Bl/6 mice, 6–7 weeks old and weighing 20-25 g were obtained from Janvier Labs (Le Genest St. Isle, France). C57Bl/6-CD11c-GFP-DTR, C57Bl/6-Lang-GFP mice (a generous gift of Dr. B. Malissen, CIML, Marseille, France) and TLR9^−/−^ mice (gift from Invivogen, Toulouse, France) were bred in our animal facility. Nude mice were purchased from Charles River Laboratories France (L’Arbresle, France). Hspa1b-LucF (+/+) Hspa1b-mPlum (+/+) mice were obtained from F. Couillaud (EA7435, Université de Bordeaux, France). Hspa1b-LucF (+/+) Hspa1b-mPlum (+/+) mice contained a transgene that allowed firefly luciferase and mPlum fluorescent protein expression under the control of thermo-inducible heat-shock protein (Hsp70) promoter 1B (Hspa1b) [36]. All experiments involving animals were approved by the ethic committee (n°APAFIS#4268-2016022516345743v2) and performed in compliance with relevant laws and institutional guidelines.

### Tumor model

For tumor implantation, 0.5 × 10^6^ B16F10 melanoma cells, in 20 μl of PBS, were injected intradermally on the shaved flank of anesthetized mice. The injection was done using a 50 μL Hamilton syringe (Hamilton CH-7402 Bonaduz, GR. Switzerland) adapted with a 29G insulin needle. Tumors were detected 3 to 5 days after implantation and tumor growth was monitored every other day with a digital caliper. Tumor volume was determined by measuring the 3 dimensions of the tumor (a, b, c) and using the following formula: Volume (mm^3^) = (π/6)*(a*b*c). Mice bearing tumors reaching a volume greater than 1200mm^3^ or necrotic tumors were sacrificed.

For the re-challenge, 0.5 × 10^6^ B16F10 cells were injected intradermally as previously described on the opposite flank of the primary tumor.

To evaluate the systemic effect of the treatment, 0.5 × 10^6^ B16F10 cells were injected intradermally the same day on both flanks of the animal. While one of the tumors was treated with IL-12 GET and pIRE the other tumor was left untreated. The size of both tumors was measured every day.

### pIRE treatment of tumor bearing mice

Partially irreversible electropermeabilization (pIRE) was performed using the GHT Uni2000 from Beta Tech (Saint-Orens, France) connected to a digitized oscilloscope Gould (DSO)1602 (Gould, Ilford-Essex-UK). The pulse profiles were displayed and stored on the oscilloscope for an on-line control of the delivery. Tumors were treated using stainless steel, flat, parallel and 0.4 cm-gap electrodes (IGEA, Capri, Italy). The tumor was squeezed between the two plate electrodes using Echogel (Comepa, St Denis, France) to obtain a good electrical contact (See Additional file 1: Figure S1). pIRE conditions consisted of 10 square waved pulses of 1200 V, duration 100 μs, delivered at a frequency of 1 kHz. High voltage and pulse duration used were classical values for IRE [18, 20]. Only the number of pulses was reduced to obtain partial IRE. The use of 1 kHz was preferred because of the shortened treatment duration inducing only one muscle contraction as already shown for ECT treatment [37]. Mice were gas-anesthetized with 2% (v/v) of isoflurane and were kept under anesthesia during the whole procedure. Mice without EP served as control group for experiments.

### In vivo fluorescence and color acquisitions of B16F10-eGFP tumors

Visualization of B16F10-eGFP in vivo transfected cells was performed over several days after pIRE treatment using a Macrofluo microscope (Leica, Wetzlar, Germany) equipped with a cooled CCD camera (Roper Coolsnap HQ, Photometrics, Tucson, AZ). Animals were kept under isoflurane anesthesia during the observation. Color imaging was obtained by use of CRI Micro*Color 2 Liquid Crystal Technology. The fluorescence excitation was obtained with an EL6000 light source (Leica, Wetzlar, Germany) and the L5 (ex = 480/40 nm, em = 527/30 nm) filter set (Chroma technology, Rockingham, USA) for GFP observation. These images allowed analysis of tumor volume and GFP expression on the same animal during several days.

### Bioluminescence imaging and measurements

The Hspa1b-LucF (+/+) Hspa1b-mPlum (+/+) mice allowed to follow up the induction of Hspa1b promoter through the expression of luciferase reporter protein which could then be imaged with bioluminescence in vivo imaging.

Values for thermal stress positive control were determined between 39 and 45 degrees Celsius as already described [34] and luciferase activity was measured in the anesthetized animal using a cooled CCD camera (iKon M, Andor, Belfast, UK) as a measure of reporter gene expression. Reporter gene expression was assessed at 6 h post-heating or pIRE treatment, 5 min after intraperitoneal injection of luciferin (3 mg/mouse) in 100 μL of PBS. Mean bioluminescence intensity in the treated area was determined using FIJI [34].

### Plasmid injection and gene-electro-transfer protocols

GET treatment was performed as described in Pasquet et al. [34]. Briefly, tumor-surrounding skin was shaved and depilated using a depilatory cream the day prior treatment. When the B16F10 tumor volume reached 7 to 15mm^3^, two injections of 25 μg of IL-12 encoding pCpGfree-mIL-12 (p35p40) plasmid (pIL-12) in 20 μL of PBS were performed in two distinct areas in the skin dermis directly surrounding the tumor (from 2 to 5 mm from the edge of the tumor). Stainless steel, contact electrodes (0.4 cm gap) with Echogel were applied on the skin around the injection point. Four trains of square-wave unipolar High voltage-Medium voltage (HV-MV) pulses were applied using Electrocell B10 generator (Leroy Biotech, St Orens, France). Trains of HV pulses consisted of 100 μs square waved pulses of 400 V. Trains of MV pulses consisted of 20 ms square waved pulses of 100 V. The delay between each pulse was 50 ms and each pulse train was applied with a 1 Hz frequency. The proper delivery of the pulses was monitored live on a touch screen. In preliminary experiments, Il-12 transfection was performed 48 h before pIRE (pIRE + IL-12 T-48 h), or the same day (pIRE + IL-12 T0 h) or 24 h after pIRE treatment (pIRE + IL-12 T + 24 h) (Additional file 2: Figure S2). The 48 h delay between GET and pIRE treatments was selected as the most effective in tumor total regression. (pIRE +IL-12 T-48 h.) (See Additional file 2: Figure S2).

### Histology

Tumors were collected at 6 h and every day from 1 to 7 days after pIRE treatment. Samples were fixed in PLP buffer (0.05 M phosphate buffer, 0.1 M lysine, 2 mg/ml sodium periodate, 1% paraformaldehyde) for 24 h. The day after, tissues were incubated 3 h in successive sucrose solutions (10, 20 and 30%) and embedded in OCT. Five micrometer thick sections were cut, stained with hematoxylin-eosin (Sigma Aldrich, France) and mounted with Mowiol mounted medium. Observation was performed with a scanner reader (Pannoramic 250, 3DHISTECH).

### Apoptosis measurements

Tumors were collected 24, 48 and 72 h after pIRE treatment. Tumors were dissociated and single cell suspensions were labelled with Annexin V-FITC (Ozyme-Biolegend, St Quentin Yvelines, France) and 7AAD to evaluate the percentage of 7AAD negative and AnnexinV positive cells i.e. percentage of apoptotic cell death, by flow cytometry (FacsCalibur, BD Biosciences).

### Determination of IFNγ production in IL-12 GET treated skin

Mice were treated with 2x25μg of IL-12 plasmid as described above. Control mice were injected with PBS and submitted to the same GET protocol. PBS GET and pIL-12 GET treated areas, as well as contralateral skin, were clearly identified and harvested 1, 2, 3, 4, 7 and 14 days post-treatment. Samples were incubated in 2 mL complete culture medium at 37 °C, 5% CO_2_ for 24 h.

IFN-γ content in the culture supernatant was determined by ELISA according to the manufacturer protocol (ELISA MAX Deluxe Set, Biolegend, San Diego, CA).

### Toxicity tests

pIL-12 GET toxicity was evaluated with hematology, biochemistry and anatomo-pathology results. The testing facility was C.RIS Pharma (Saint Malo, FRANCE) (Study No.: CP-2016029).

Two injections of 25 μg of plasmid in 20 μL of PBS were performed in two distinct areas in the dermis of the skin. Four trains of square-wave unipolar High voltage-Medium voltage (HV-MV) pulses were applied. Toxicity was performed at D15 and D49 after the GET.

Hematological parameters (white blood cells, hemoglobin, red blood cells, hematocrit, mean corpuscular volume, platelets, mean corpuscular hemoglobin, mean corpuscular hemoglobin concentration) were determined on 2 female and 2 male mice treated with control and 4 female and 3 male mice treated with GET sacrificed at D15 and on 2 female and 2 male mice treated with control and 4 female and 4 male mice treated with GET sacrificed at D49.

Biochemistry parameters (glucose, urea, creatinin, triglycerides, cholesterol, protein, albumin, GOT, GPT, calcium, phosphore, chloride, sodium, potassium, bilirubin) were determined on 2 female and 2 male mice treated with control and 4 female and 3 male mice treated with GET sacrificed at D15 and on 2 female and 2 male mice treated with control and 4 female and 4 male mice treated with GET sacrificed at D49.

### Statistical analysis

Statistical analysis was carried out using Prism 5 statistical software (GraphPad Software Inc., San Diego, CA). Values are means ± SEM. We used linear regression, 2way ANOVA t-test analysis. We used Log-rank (Mantel-Cox) test for survival curves.

## Results

### pIRE parameters reduced tumor growth and increased survival

**Fig. 1:**
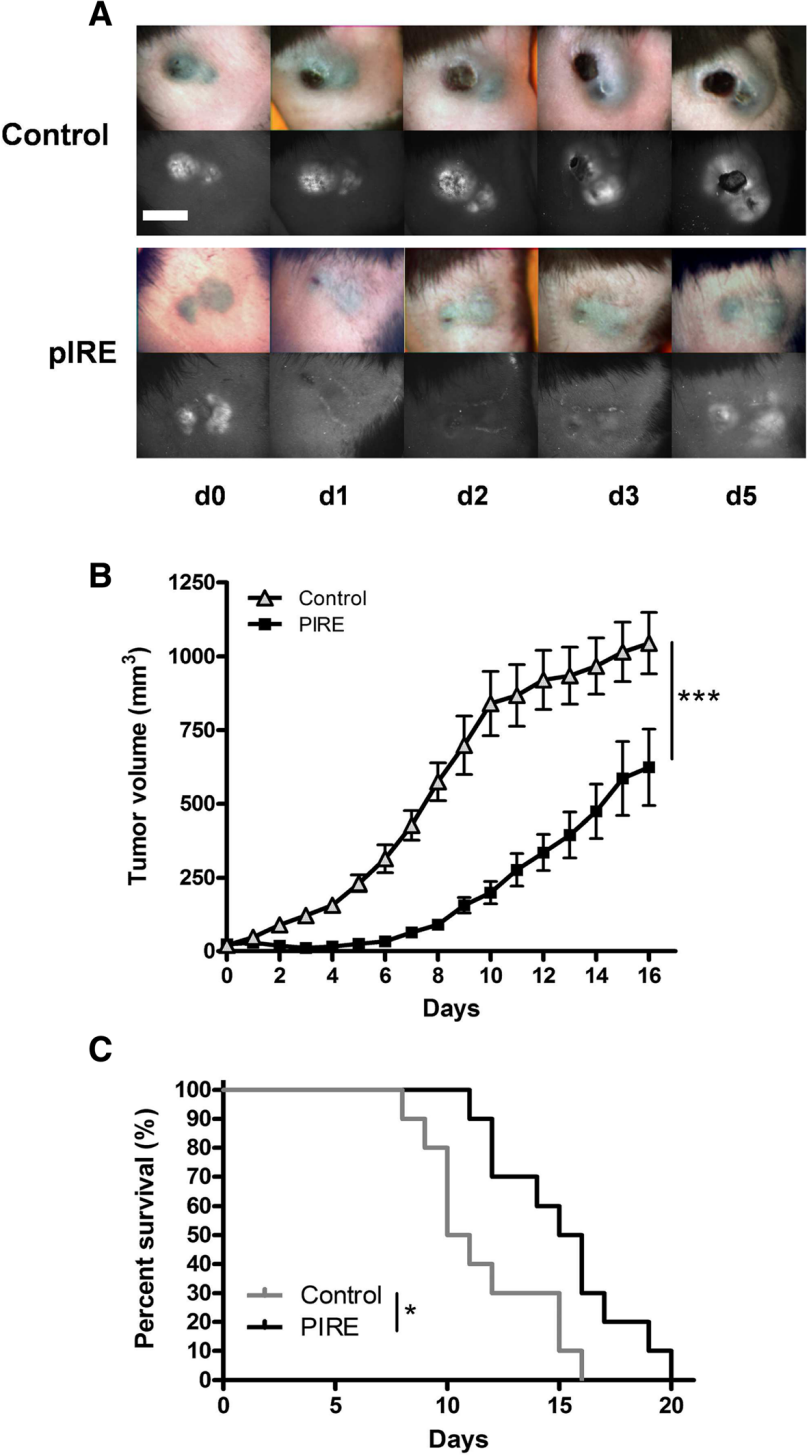
Effect of pIRE treatment on tumor growth and survival. C57Bl/6 mice were intradermally injected with 0.5 × 106 B16F10-GFP tumor cells and treated with pIRE parameters: 10 square waved pulses of 1200 V, duration 100 μs, frequency 1 kHz. Tumor volume was followed up every day (d) post treatments with a digital caliper and GFP expression was monitored in vivo. **a** Macroscopic visualization of tumor size and GFP expression following pIRE treatment. Scale bar represents 5 mm. **b** Tumor growth representation of untreated tumors (Δ) and pIRE treated tumors (■). Values are means ± SEM. ***P < 0.001, (Two-way ANOVA analysis). **c** Kaplan-Meyer survival curve of untreated mice (gray line) and pIRE treated mice (black line). *N* = 10 mice per group. (*P < 0.05 (Log-rank (Mantel-Cox) Test)

In order to mimic the situation where irreversible electropermeabilization did not bring a complete eradication of the tumor, we first delivered PEF with parameters inducing a partial-irreversible electropermeabilization (pIRE) of melanoma. C57Bl/6 mice were intradermally injected with 0.5 × 10^6^ B16F10-GFP cells and treated or not with pIRE parameters. Without any treatment, tumors had a fast growth rate and all mice had to be sacrificed maximum 16 days after tumor induction (Fig. 1). Compared to untreated tumors, pIRE treatment transiently reduced tumor size in the first five days after treatment inducing a delay in tumor growth (Fig. 1a and b). During this 5 days period, GFP fluorescence was not detectable in pIRE treated tumors showing a transient knock-down in cellular metabolism (Fig. 1a). Therefore, pIRE treatment significantly increased survival of mice compared to untreated mice (Fig. 1c). Similar delays in tumor growth and survival were obtained on different mouse strains (See Additional file 3: Figure. S3).

### pIRE treatment induced tumor cell death

**Fig. 2:**
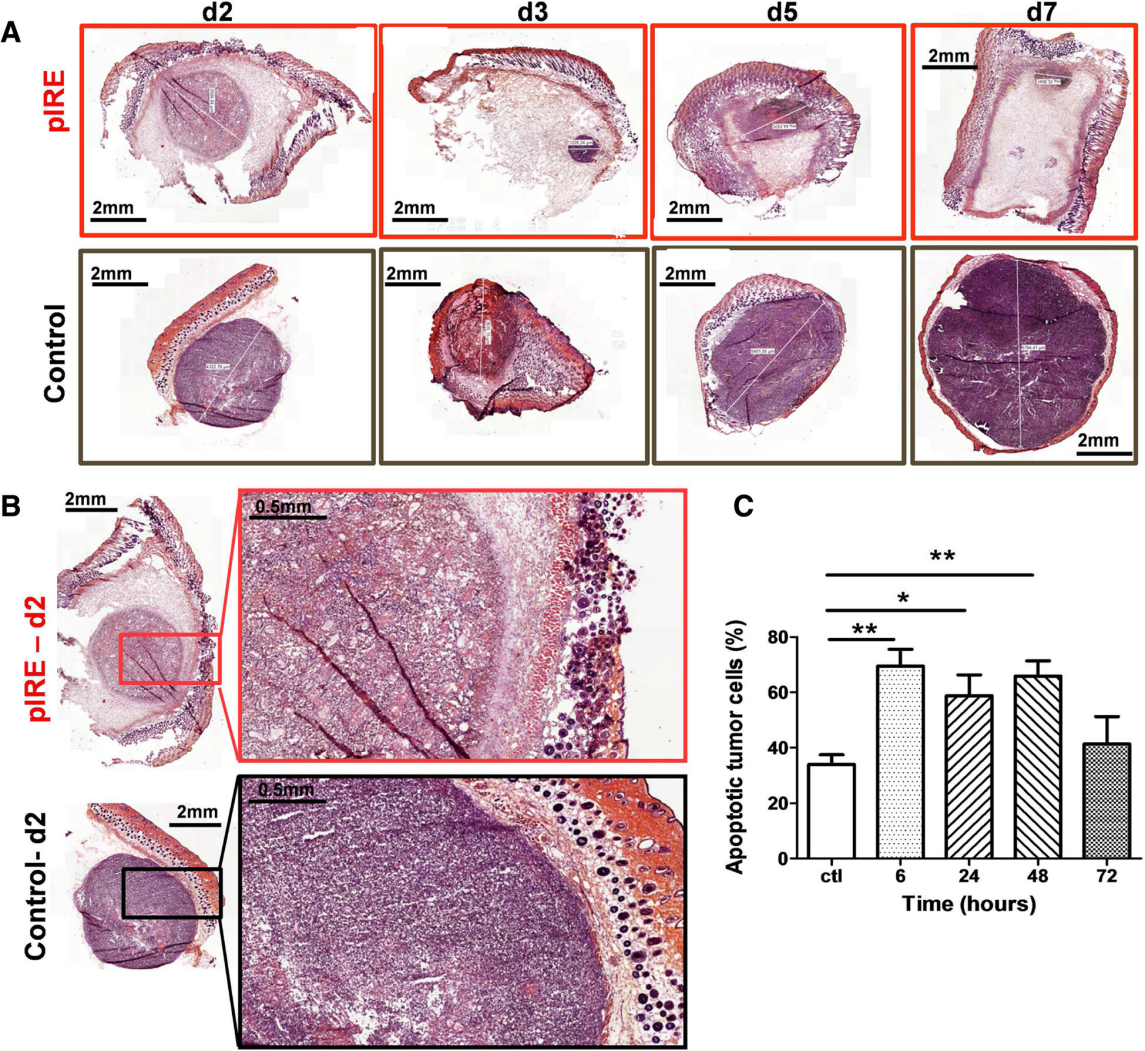
pIRE induced tumor apoptotic cell death. C57Bl/6 mice were intradermally injected with 0.5 × 106 B16F10 cells. When the tumor reached a volume of 20 to 30 mm3, pIRE parameters were applied: 10 square waved pulses of 1200 V, duration 100 μs, frequency 1 kHz. A and B-Tumor were collected from 2 to 7 days after pIRE treatment. After fixation and inclusion, 5 μm sections were cut, stained with eosin and hematoxylin, mounted and observed with a scanner. **a** Eosin-hematoxylin representative images of skin tumors 2 (d2), 3 (d3), 5 (d5) and 7 days (d7) after pIRE treatment (up line) or without treatment (bottom line). Tumor diameter is highlighted by a white line. **b** High magnification, 2 days after pIRE, of representative images of skin tumors treated (top) or not (bottom). **c** Tumors were collected 6, 24, 48 and 72 h after pIRE treatment, dissociated, and cells suspensions labelled with Annexin V-FITC and 7AAD to characterize cell death. Histograms of Annexin V positive/7AAD negative cells described as early apoptotic in untreated mice (CTL) and 6 to 72 h post pIRE treatment. For untreated mice, a tumor from each time point was collected. Values are means ± SEM of 4 mice. * < 0.05 and **P < 0.01 (Two-way ANOVA analysis)

In order to characterize the effect of pIRE treatment on the tumor, we observed the microscopic evolution of treated tumors by histology 2 to 7 days after pIRE treatment (Fig. 2a). Treated and untreated tumors of identical initial volumes were collected 2, 3, 5 and 7 days after pIRE treatment and stained with hematoxylin and eosin. The size of treated tumors appeared significantly reduced 3 days after treatment compared to untreated tumors (Fig. 2a). Moreover, while untreated tumors grew quickly, the size of pIRE treated tumors remained overall stable from day 3 to 7 even though still measurable. The observation at a higher magnification 2 days after the treatment revealed a reduced cellularity in the treated tumors observable by the presence of “holes” inside the tumors (Fig. 2b). These blank areas were a proof of tumor cell death induced by pIRE treatment.

In order to better characterize the cellular events, we harvested treated tumors 6 to 72 h after treatment and stained the cells with Annexin V and AAD7 to distinguish apoptosis and necrosis pathways (Fig. 2c). A positive staining with 7AAD revealed a loss of cell membrane integrity, whereas the flip of phosphatidylserine, an early event characteristic of apoptosis, was identified by a solo Annexin V staining. Early apoptosis (Annexin V+/ 7AAD-) was mostly present during two days after pIRE treatment showing a 2-fold increase of apoptotic cells relative to control tumors. At 72 h after treatment when the tumor size reached its minimum value, the percentage of apoptotic cells was not significantly different from control tumors.

### pIRE treatment did not induced any thermal side effects

To study the role of thermal effect in pIRE induced tumor cell death, the pIRE procedure was applied on tumors implanted in Hspa1b-LucF (+/+) Hspa1b-mPlum (+/+) mice and followed by non-invasive bioluminescence imaging. A slight luminescent signal was observed in the treated zone. The positioning of the electrodes was visualized 6 h after treatment (See Additional file 4: Figure S4A) and the mean bioluminescence intensity was quantified showing a significant increase when compared to untreated tumor (See Additional file 4: Figure S4B). The ratio of mean bioluminescence intensities between the treated and untreated areas showed a 1.6+/− 0.3 fold increase (See Additional file 4: Figure S4C). The corresponding increase of temperature in the treated zone was determined using a positive control for thermal stress and corresponded to the 8 min exposition of the control mice to 39–40 °C. (See Additional file 4: Figure S4D). This low temperature was the experimental evidence that pIRE treatment did not induce thermal side effect and that the pIRE induced tumor cell death was not due to a thermal process but was a consequence of the electroporation induced membrane disruption.

In conclusion, the PEF parameters we set induced a partially irreversible electroporation (pIRE) by causing apoptotic tumor cell death resulting in transient tumor regression. Importantly, this treatment did not induce immediate side effects. However, after 5 days, treated tumors seemed to grow at the same rate as untreated tumors suggesting no visible implication of the adaptive immune system. Nevertheless, hidden tumor antigens might have been released during the 2 days following the pulse delivery. Therefore, we tested the boosting effect of IL-12 cytokine expression on the antitumor immune response.

#### pIL-12 GET followed by pIRE induced tumor regression

**Fig. 3:**
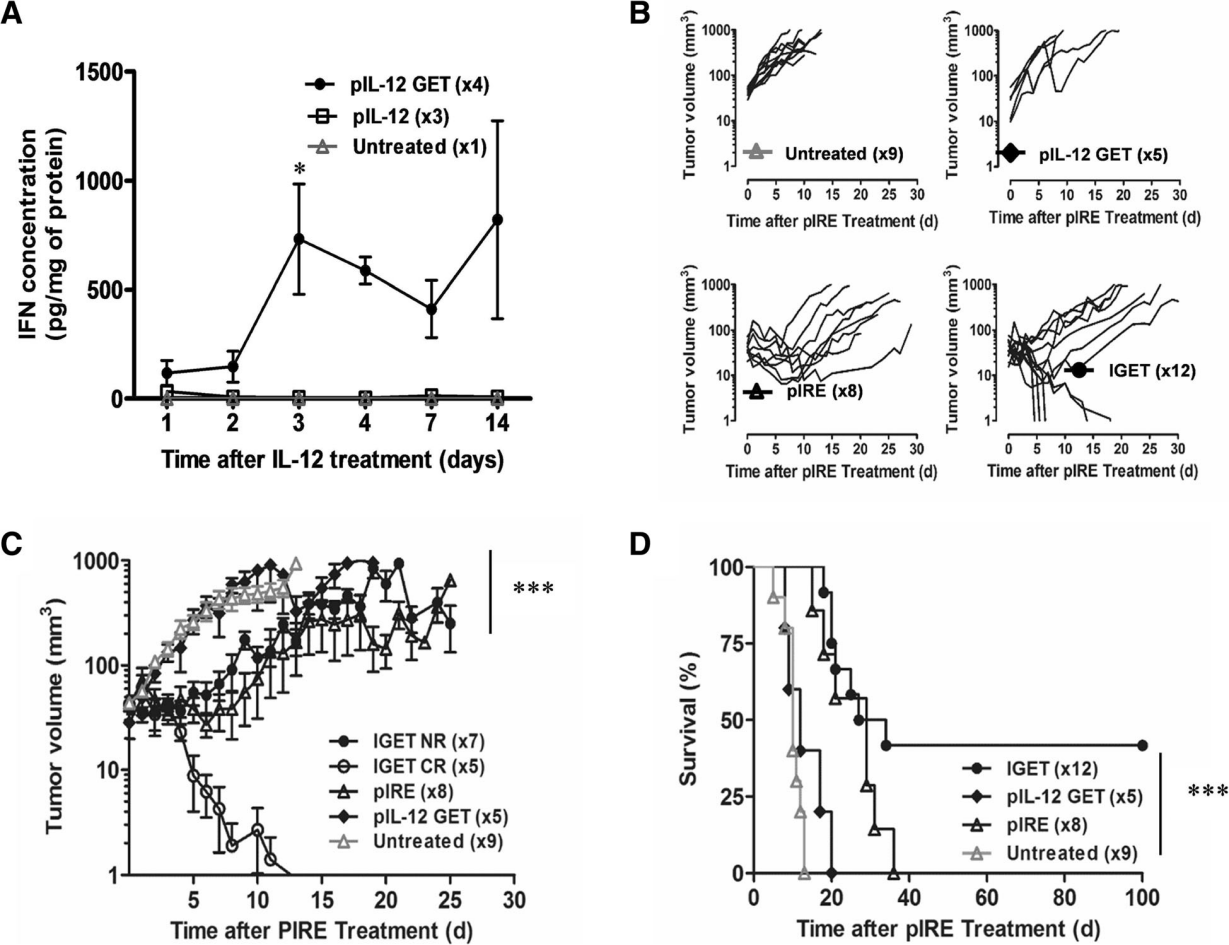
IGET treatment induced partial B16F10 tumor regression. **a** CD11c-GFP-DTR mice were intradermally injected with 25 μg of pIL-12 in 20 μl PBS, or with 20 μl of PBS alone in one flank and were submitted to GET protocol. The contralateral flank from IL-12 injected mice was non injected and non-pulsed to be used as an internal control. Mice were sacrificed from 1 to 14 days after treatment and skin from each flank were harvested and cultured overnight in complete medium 5%CO2, 37 °C. IFN-γ content was determined in the cultured supernatant of pIL-12 treated skin (pIL-12 GET), of pIL-12 untreated skin (pIL-12) and of PBS treated skin (PBS GET) by ELISA. 3 ≤ *n* ≤ 4, 2 independent experiments, statistical analysis: 2way Anova, *p < 0.05. **b** CD11c-GFP-DTR mice were intradermally injected with 0.5 × 106 B16F10 cells. When the tumor reached a volume of 7 to 15 mm3, 2x25μg of pIL-12 were injected intradermally in the healthy skin directly surrounded the tumor. Electrotransfection was realized using GET protocol. Two days later, pIRE parameters were applied on previously treated tumors. Tumor volume was followed up to 100 days in case of complete regression. Individual curves are plotted for untreated control, pIL-12-GET alone, pIRE alone, and IGET treated mice. **c** Mean +/− SEM tumor growth progression while separating IGET treated group between complete regression group (CR) and non-responding group (NR). From day 4, ***p < 0.001 between IGET CR and untreated or pIL-12-GET groups (Two-way ANOVA analysis). **d** Mice survival of untreated control, pIL-12 GET, pIRE and IGET treated tumor. ***P < 0.001 (Log-rank (Mantel-Cox) Test) between untreated control, pIL-12 GET, pIRE and IGET treated tumor (5 ≤ *n* ≤ 12, 3 independent experiments)

We recently showed that IL-12 expression was significantly increased following GET protocol [34]. Here, we verified that IL-12 expression can induce IFN-γ production (Fig. 3a). A significant amount of IFN-γ was quantified locally, only when pIL-12 GET was performed and not in the untreated contralateral skin.

To test the efficacy of pIL-12 GET combined with pIRE pulses on regression of skin tumors, C57Bl/6 mice were intradermally injected with 0.5 × 10^6^ B16F10 cells. In absence of treatment, tumors grew quickly and all mice had to be sacrificed maximum 13 days after tumor induction (Fig. 3b-d). One group of mice was treated with pIL-12 peritumoral GET which did not induce any significant difference on B16F10 tumor growth (Fig. 3b, c) or survival of mice (Fig. 3d) compared to untreated control mice. As we previously described, pIRE parameters induced a transient tumor shrinkage (Fig. 3b, c) and therefore an increase in survival of mice for up to 15 days compared to untreated animals (Fig. 3d). The last group of mice treated with pIL-12 GET and 2 days later with pIRE displayed two different tumor behaviors. Seven out of 12 mice never rejected the tumor and displayed the same growth rate as pIRE treated mice (Fig. 3b, c). However, 5 out of 12 mice had totally rejected the tumor (41.6% of complete regression) (Fig. 3b, c). This regression lasted for 100 days after treatment (Fig. 3d). One of the mouse needed to be sacrificed because of teeth malformation that could no longer be cured. Nevertheless, the combination of pIL-12 GET and pIRE not only enhanced survival but could bring a curative effect. This two-step treatment was named Immune-Gene Electro-Therapy (IGET).

#### IGET induced memory immune response

**Fig. 4:**
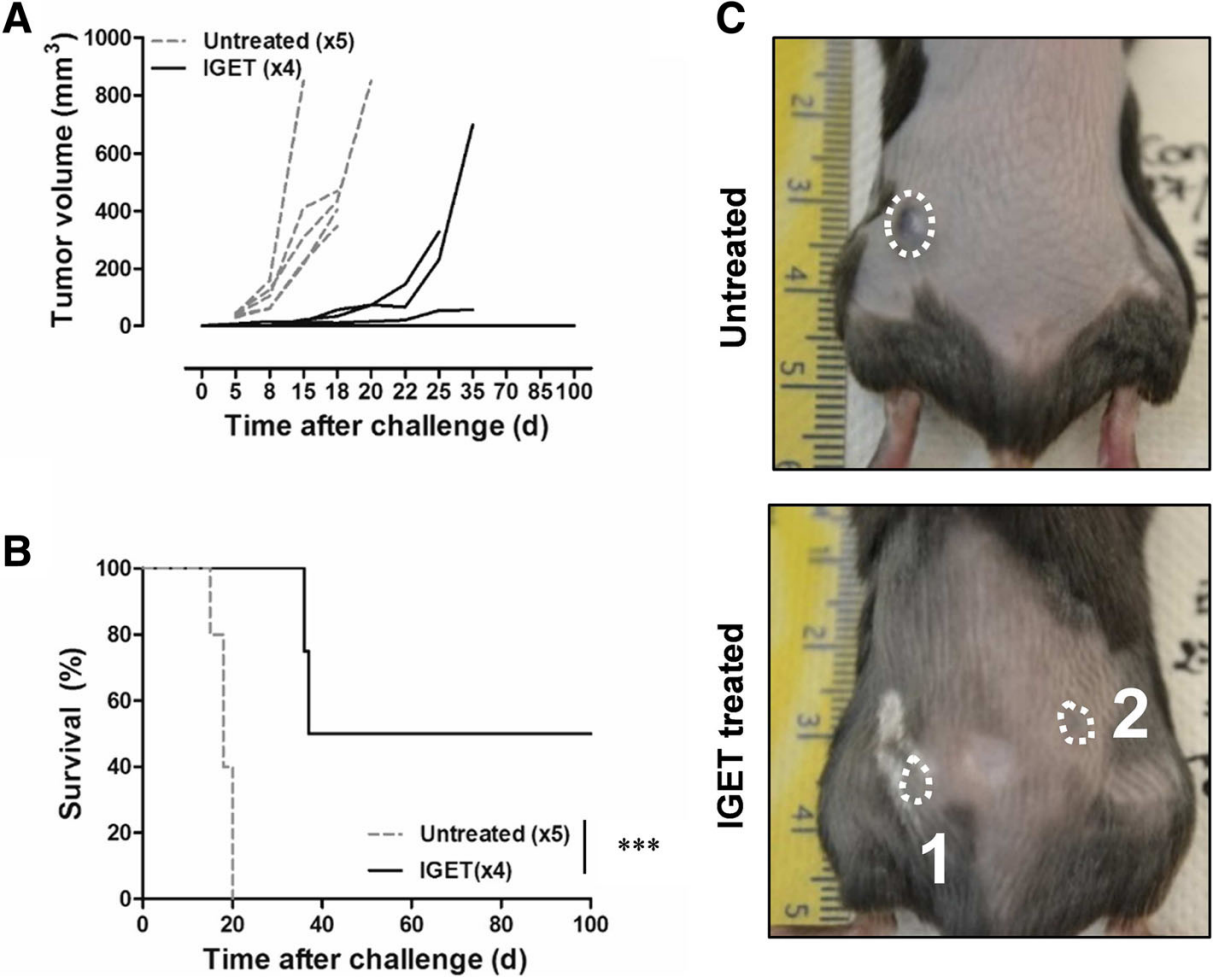
IGET induced a memory immune response. Mice experiencing complete regression of the tumor 100 days after the first treatment were injected again with 0.5 × 106 B16F10 cells. Injection in naïve mice served as control (untreated). Tumor growth was followed up to 100 days. **a** In IGET treated group (black line), 2 mice developed the tumor, 1 needed to be sacrificed for external purpose, 2 mice never developed tumors. **b** Mice survival in untreated (dash gray line) and IGET treated group (black line), ***P < 0.001 (Log-rank (Mantel-Cox) Test) (**c**) Pictures illustrating the position of injection sites and the absence of tumor development in an IGET mouse (bottom). 4 ≤ *N* ≤ 5 mice per group

Mice with complete tumor regression were injected with 0.5 × 10^6^ B16F10 cells, in the opposite flank to the first tumor, 100 days after the IGET treatment. Control naïve mice developed tumors in 3 to 5 days and all mice had to be sacrificed between 18 and 20 days after tumor induction (Fig. 4). Among the four challenged mice, 2 developed a tumor at the new site of implantation, but with a delay of 15 days compared with untreated mice (Fig. 4a). Interestingly, 2 out of 4 challenged mice never developed tumor up to 100 days after the second induction (Fig. 4). In conclusion, the IGET treatment induced a long-term memory immune response controlling the development of distant new tumors.

#### IGET treatment slowed down distant tumor growth

**Fig. 5:**
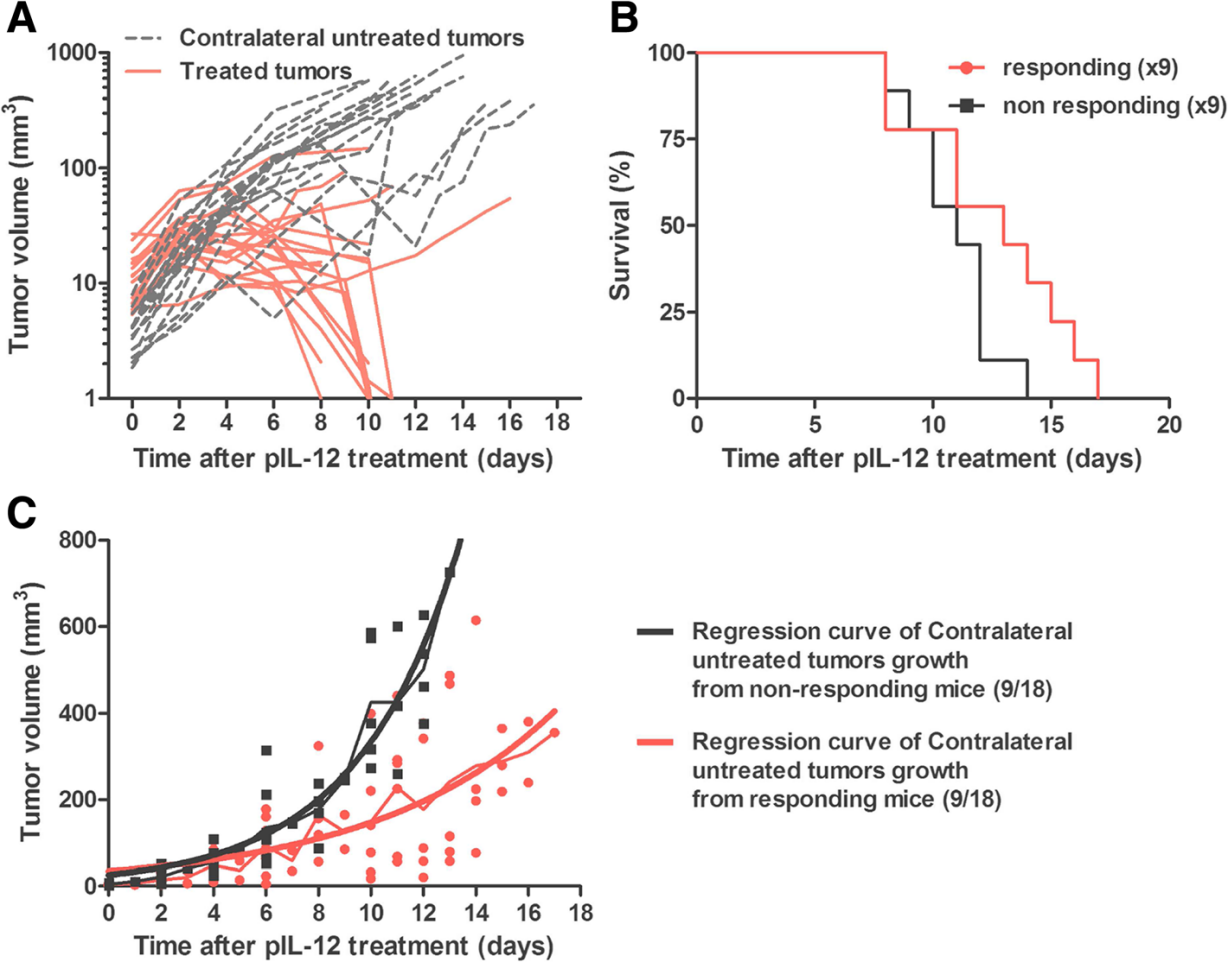
IGET treatment slowed down distant tumor growth. CD11c-GFP-DTR mice were intradermally injected with 0.5 × 106 B16F10 cells in both flanks. When one of the tumors reached a volume of 7 to 15 mm3, 2x25μg of pIL-12 were injected intradermally in the healthy skin directly surrounding the tumor. Electrotransfection was realized using HV-MV electrical parameters. Two days later, pIRE parameters were applied on previously treated tumor. **a** The volume of treated (red) and untreated (black) tumors was measured until one of the tumor reached 1200 mm3. As described previously treated tumors can be divided in responders (rejection of the treated tumor) and non-responders (no rejection of the treated tumor), (**b**) Survival curves of responding and non-responding mice and (**c**) growth curves of untreated tumors from responding mice (red) and non-responding mice (Black). Dots represents individual points; thin lines, the median of tumor growth; thick lines, the calculated regression curves. *N* = 18, 2 independent experiments

In the clinic, treated patients frequently have several nodules in the same area. We have previously demonstrated that the induced IL-12 production is specifically localized at the site of the electro-gene-transfer [34]. Therefore, to analyze the efficacy of the IGET on distant tumors, B6 mice were injected on both flanks with B16F10 cells. IGET was applied only on one developing tumor. As described in Fig. 3, two different behaviors were observed. 50% of the treated mice (9 out of 18) presented a tumor regression on the treated tumor, while the other 50% (9 out of 18) presented no tumor regression (Fig. 5a). The mice with regression were identified as responding mice and the others as non-responding mice. No regression of contralateral non-treated tumors was detected on responding or non-responding mice. Despite a non-statistically significant difference (Log-rank (Mantel-Cox) test; *p* value = 0.0983), we observed a slightly increased survival of responding mice compared to non-responding mice (median survival of 13 and 11 days respectively) (Fig. 5b). We attributed this result to a difference in growth rate of distant tumors. Therefore, we analyzed separately the growth rate of distant tumors from responding and non-responding mice by comparing the tau value of exponential growth regression curve associated to each group (Fig. 5c). The distant tumor tau value from responding mice (3.907, R^2^ = 0.4687) was lower (about 56%) than the one from non-responding mice (6.899, R^2^ = 0.8819). This result suggested a role of the immune system, modulated by IL-12 expression, controlling tumor development at the distant site.

#### IGET induced tumor regression depended on adaptive immune response

**Fig. 6:**
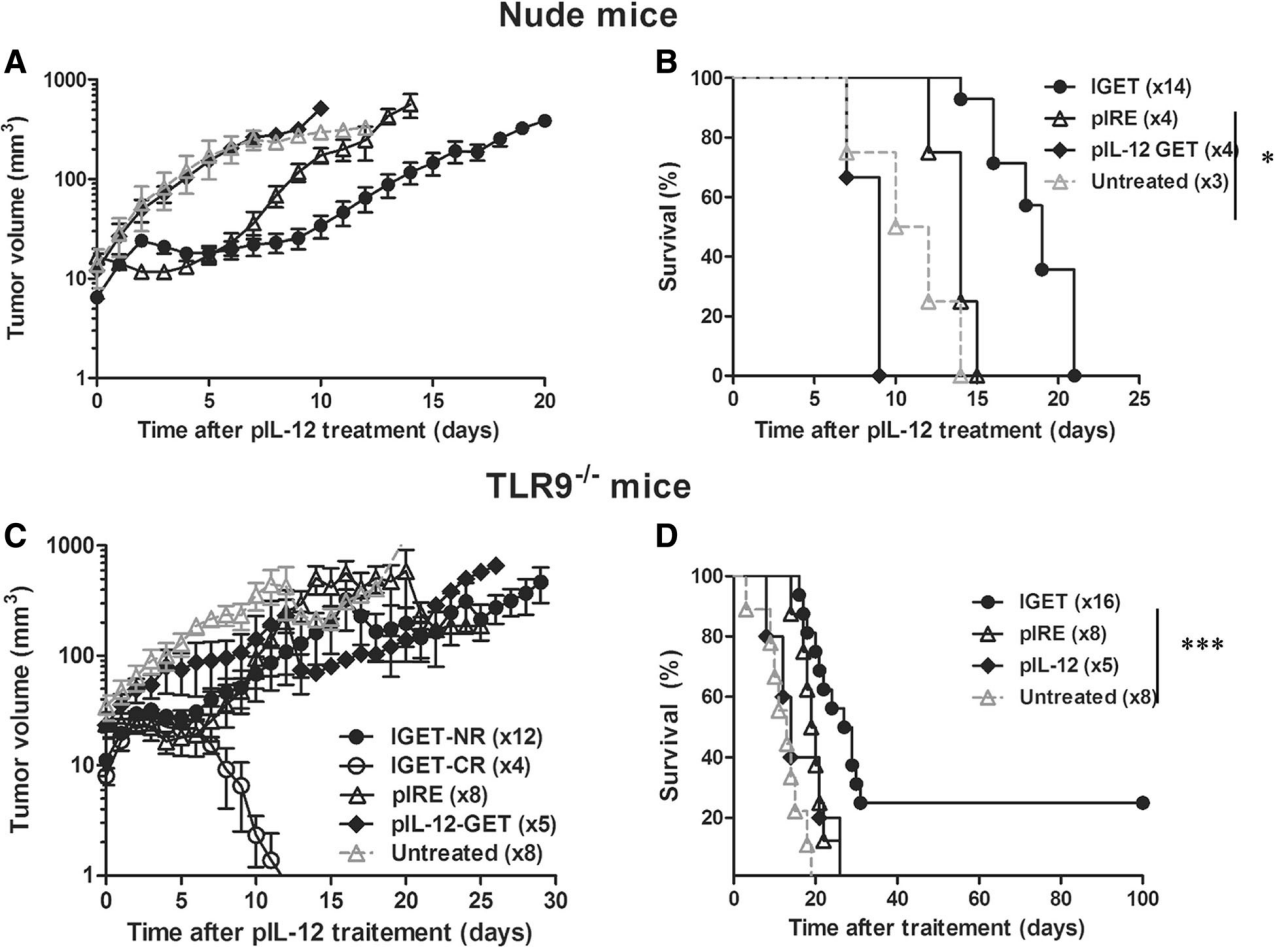
IGET induced tumor regression depended on adaptive immune response and not due to a direct effect of plasmid DNA. Nude mice (**a** and **b**) and TLR9−/− mice (**c** and **d**) were intradermally injected with 0.5 × 10^6^ B16F10 cells. When the tumor reached a volume of 7 to 15 mm3, 2x25μg of pIL-12 were injected intradermally in the healthy skin directly surrounding the tumor. Electrotransfection was realized using HV-MV electrical parameters. Two days later, pIRE parameters were applied on previously treated tumors. As control, mice were only treated with pIL-12 GET, pIRE or untreated. Tumor volume (**a**, **c**) and mice survival (**b**, **d**) was followed until tumor volume reached 1200 mm^3^. *p < 0.05, ***P < 0.001 (Log-rank (Mantel-Cox) Test between untreated control, pIL-12 GET, pIRE and IGET treated tumor. The number of mice is indicated on the graph in 2 independent experiments

To analyze the putative role of the immune system in the tumor growth control, IGET protocol was applied to nude immunodeficient mice implanted with 0.5 × 10^6^ B16F10 cells. Both pIRE and IGET treated mice presented a transient regression of tumor volume (Fig. 6a). But importantly, no complete regression could be observed among IGET treated nude mice (Fig. 6a) contrary to WT mice (Fig. 3b, c) suggesting an implication of the adaptive immune system. Nevertheless, IGET treated tumors in nude mice presented a slower growth rate than pIRE treated or untreated mice resulting in increased survival (Fig. 6b). This result suggested an implication of other mechanisms controlling tumor growth. Despite that, all (14 out of 14) IGET treated nude mice were sacrificed between 15 and 22 days after treatment compared to 42% of complete regression in WT mice.

#### IGET induced tumor rejection implicated innate immunity

In this protocol, naked DNA was injected intradermally before pIRE was applied. Internalized naked DNA recognized by the Toll Like Receptor 9 (TLR9) can directly activate immune cells. We used CpG free constructions to avoid this problem. Nevertheless to understand whether the positive effect observed with this treatment implicated a bystander effect by the introduction of foreign DNA, TLR9^−/−^ mice were intradermally injected with 0.5 × 10^6^ B16F10 cells and then submitted to IGET. Interestingly, TLR9^−/−^ mice resumed the bimodal response observed in WT mice (Fig. 6). Among IGET treated TLR9^−/−^ mice, 4 out of 16 (25%) experienced a complete regression lasting 100 days after the treatment (Fig. 6c, d).

In conclusion, even though the efficacy of the treatment was reduced in mice with an impaired innate immunity, it appeared that the positive effect obtained with IGET treatment implicated involvement of other mechanisms not only the recognition of naked pDNA by innate receptors.

#### Toxicity of the pIL-12 GET

The analysis of hematological parameters showed no difference between control groups and GET groups for male and female mice at D49. Few differences were observed at D15, probably due to sample coagulation but not to a toxicity of the treatment (Table 1).

**Table 1:** Hematology analysis of male and female mice of both groups at D15 and D49

Female mice		D15		D49	
		Group 1	Group 2	Group 1	Group 2
Parameters	Unity	Control	EGT	Control	EGT
White blood cells	10^3/μl	6.4 ± 1.7	2.9 ± 1.5	7.7 ± 2.1	6.3 ± 1.0
hemoglobin	g/dl	13.0 ± 0.3	10.9 ± 3.5	13.8 ± 1.1	14.0 ± 1.3
Red blood cells	10^6/μl	5.5 ± 3.2	6.7 ± 2.1	8.4 ± 0.8	8.4 ± 0.7
Hematocrit	%	26.6 ± 15.3	32.9 ± 11.6	40.6 ± 4.2	40.8 ± 3.9
Mean corpuscular volume	fl	48.7 ± 0.2	48.5 ± 2.6	48.3 ± 0.3	48.4 ± 0.7
Platelets	10^3/μl	333 ± 180	536 ± 297	376 ± 6	373.8 ± 62.5
Mean corpuscular hemoglobin	pg	28.3 ± 16.0	16.2 ± 1.5	16.4 ± 0.3	16.5 ± 0.3
Mean corpuscular hemoglobin concentration	g/dl	58.4 ± 32.7	33.6 ± 4.0	34.0 ± 0.7	34.2 ± 0.1
Male mice		D15		D49	
		Group 1	Group 2	Group 1	Group 2
Parameters	Unity	Control	EGT	Control	EGT
White blood cells	10^3/μl	5.6 ± 2.3	8.0 ± 1.9	8.4 ± 0.1	7.5 ± 3.4
hemoglobin	g/dl	13.0 ± 2.5	14.9 ± 1.0	13.9 ± 1.4	13.2 ± 0.7
Red blood cells	10^6/μl	7.6 ± 1.5	8.6 ± 0.6	8.3 ± 1.1	8.0 ± 0.3
Hematocrit	%	36.8 ± 7.3	42.5 ± 1.9	40.9 ± 3.5	38.7 ± 1.3
Mean corpuscular volume	fl	48.5 ± 0.1	49.6 ± 1.2	49.6 ± 2.6	48.3 ± 0.6
Platelets	10^3/μl	351 ± 6	345 ± 112	424 ± 62	368 ± 61
Mean corpuscular hemoglobin	pg	17.0 ± 0.1	17.3 ± 0.4	16.8 ± 0.6	16.4 ± 0.3
Mean corpuscular hemoglobin concentration	g/dl	35.2 ± 0.3	34.9 ± 1.1	34.0 ± 0.6	34.0 ± 1.1

2x25μg of pIL-12 were injected intradermally in the healthy skin of female and male C57Bl/6 mice. Electrotransfection was realized using HV-MV electrical parameters. Hematological parameters (white blood cells, hemoglobin, red blood cells, hematocrit, mean corpuscular volume, platelets, mean corpuscular hemoglobin, mean corpuscular hemoglobin concentration) were determined on 2 female and 2 male mice treated with control and 4 female and 3 male mice treated with GET sacrificed at D15 and on 2 female and 2 male mice treated with control and 4 female and 4 male mice treated with GET sacrificed at D49. Values represent mean ± SD

The analysis of biochemistry parameters revealed no difference between control group and GET group for male and female mice at D15 and at D49 (See Additional file 5: Table S1).

Anatomopathology results were the final observations. In treated mice euthanized on day 15, diffuse chronic dermal inflammation was observed in the skin. It affected 3/3 males, with a moderate severity, and 4/4 females with a marked severity. Controls were not affected. On day 49, 1/2 control males also presented dermal inflammation with a minimal severity. 3/4 treated males were affected, with a slight or moderate severity, while 2/4 treated females were also affected, with a minimal or moderate severity. Altogether, this indicated that GET induced inflammation in the dermis, which tended to disappear over time (partial recovery within 50 days). There were no systemic change related to GET at any time point. All observations were spontaneous. Under the conditions of this experiment, a single intradermal injection and skin electrotransfer administration of the CpG free construct to mice induced inflammatory changes in the skin that tended to regress over time (partial recovery).

## Discussion

In this study, we showed that peritumoral plasmid IL-12 electrotransfer followed with partially Irreversible Electropermeabilization (IGET) induced tumor regression in a mouse model of melanoma correlating with a local secretion of IL-12 [34] and IFN-γ. This treatment induced a systemic activation of the adaptive immune system leading to a growth delay of distant tumors and to the development of an immune memory that protected against the emergence of a second tumor.

When peritumoral GET of plasmid IL-12 alone was performed, we did not observe any tumor regression. Complete regression (42%) of treated tumors was observed when IL-12 GET was associated with pIRE treatment. This result could be due to the fact that peritumoral IL-12 GET did not impact the integrity of the tumor structure. The B16F10 melanoma model is a low immunogenic tumor model with low tumor-antigen spreading. Under our protocol IL-12 secretion by transfected cells failed to activate immune cells for which the contact with tumor antigens was limited. In contrast, the application of pIRE treatment, inducing destruction of tumor cells, may release tumor antigens and alarmines, danger signals released by necrotic and apoptotic cells known to activate the immune system [27, 28, 38]. In this situation, IL-12 secreted by transfected peritumoral skin cells boosted the activation of skin immune cells initiated by alarmines and recognition of tumor antigens. The efficacy of intratumoral and peritumoral IL-12 electrotransfection has been compared in a murine model of sarcoma using plate electrodes [9]. In this study, intratumoral GET induced 90% of complete regression whereas peritumoral GET induced 20% of complete regression. The type of tumor (sarcoma vs melanoma) and the distance of the peritumoral treatment to the tumor could be the reason for the difference compared to our results [6]. The same authors also demonstrated, in a mouse model of metastatic melanoma, that intramuscular electrotransfection of a plasmid encoding the integrin receptor antagonist AMEP is inefficient compared to the very efficient tumoral electrotransfection [39]. They also showed the inefficiency of intramuscular IL-12 GET alone to induce regression of a mouse model of sarcoma [9]. In this model, a positive effect of IL-12 GET was observed only when several treatments were performed combined with intratumoral electrochemotherapy (ECT).

Shirley et al. performed an impressive comparative work [40]. They not only compared the efficiency of 3 different electrical protocols but they also compared 2 different electrodes, caliper and peritumoral needles, on the transfection efficiency of a common (pUMC3-mIL-12) and an optimized (pAG250) IL-12 plasmid known to induce a higher production of IL-12. Tumors received three deliveries of pDNA on days 0, 4 and 7, while only one single pIL-12 GET was applied in the present study. The protocol bringing the highest level of IL-12 expression was highly destructive for the tumor. Shirley et al. further demonstrated that the dose of secreted IL-12 was determinant for the anti-tumor efficacy of the treatment, i.e., a lower dose of IL-12 had a more efficient anti-tumor effect than a higher dose. Interestingly, 100% of complete regression was obtained with one of the strongest electric fields applied with needle electrodes inducing important cellular damages and the lowest IL-12 production compared with the effect obtained with the other electrical parameters. By comparing studies using IL-12 GET with intratumoral, peritumoral or intramuscular injection, it appeared that, in all cases, loss of the tumor integrity was necessary to obtain an efficient action of IL-12. In our study, this was observed with the application of pIRE 2 days after IL-12 GET. In the case of intramuscular injection, this effect was obtained with the associated ECT [9]. When the IL-12 GET was performed with intratumoral injection, Shirley et al. demonstrated that the use of peritumoral needle electrodes induced local tumor damage that was equivalent to the damage obtained with pIRE treatment. Thus, our two-step modality treatment allowed us to decipher the mechanisms that might be implicated in tumor regression induced by intratumoral injection: first, the release of tumor antigens and danger signals by lysed tumor cells, and second, the full activation of several subtypes of immune cells by IL-12 secreted by transfected skin cells.

Under the conditions of this experiment, no toxicity of treatment was noted through hematology and biochemistry analyses for male or female mice at D15 and D49. A single intradermal injection and skin electrotransfer of the CpG free construct induced inflammatory changes in the skin that tended to regress over time (partial recovery). Similar observations were obtained by other groups using different plasmids and electrical pulsing parameters [41, 42].

The efficacy of IL-12 GET, whatever the site of injection, was also dependent on the number of treatments. With only one IL-12 GET and one pIRE treatment, we obtained 42% of complete regression whereas the 100% obtained in the study of Shirley et al. with peritumoral needle electrodes required 3 successive treatments [40]. Based on these results, we could further optimize the number of treatments in order to increase the efficacy of our protocol.

One of the challenges of immune therapies is to induce an efficient memory immune response. Our results showed that 50% of cured mice after IGET treatment were protected from the development of a new tumor. This was consistent with results obtained in different tumor models with different sites of injection [9, 40, 43]. A memory immune response was induced only when adaptive immune cells were activated. Treatment of nude mice showed that these T-cell-deficient mice were not able to reject the tumor, supporting the full dependence of IGET treatment efficacy on T cell activation. Nevertheless, after day 6, a sustained delay in tumor growth of IGET treated tumors was observed compared with pIRE only treated tumors. This delay could be explained by the role of IL-12 on innate immune cells. A role for natural killer (NK) cells was previously suggested [40]. Another potentially involved innate T cell population was the invariant Natural Killer T (iNKT) cell population [44–46]. These cells were specific for lipid antigens presented by the non-classical MHC molecule CD1d. iNKT cells were defined as fast producers of large amounts of IFN-γ in the hours after stimulation either directly by antigens or after interaction with APCs. Moreover, nude mice were deficient in T cells but not in B cells, and it was known that Ig production influences tumor growth [47, 48].

Another challenge of immune therapies was the induction of systemic effects in order to eliminate metastasis as well as treated tumors. Although the present single shot IGET treatment did not induce complete regression of distant tumors, their growth rate was reduced compared with untreated tumors. This observation also suggested a systemic activation of the immune system. Therefore, one could hypothesize that the electric field activated skin dendritic cells that, as a result, became more sensitive to IL-12 and more efficient in the capture of tumor antigens. Clinical development of IL-12 GET have been reported either on human [8, 49] and veterinarian [14, 50] applications.

Moreover, it was shown, using different electrical parameters and devices, that epidermal dendritic cells could be electrotransfected in vivo with plasmids encoding the GFP [51] or viral peptides and then migrated to the draining lymph node [52].

## Conclusions

Our approach appeared of interest when irreversible electropermeabilization did not bring a complete eradication of the tumor, to boost the immune system and eradicate remaining tumor cells that were not affected or were reversibly electropermeabilized. Our findings showed that IGET treatment could induce a systemic and long-lasting immunity resulting in complete regression of melanoma in mice.

This two-step procedure allowed us to further distinguish the impact of pIL-12 skin electrotransfection on one hand and of the electropermeabilized tumor on the other hand on the activation of the immune system. Therefore, our study pointed out that tumor disruption and release of tumor antigens were essential to obtain an efficient activation of the adaptive immune system. This was a hidden aspect of the protocol with peritumoral needle electrodes. Although the level and the lifetime of expression of IL-12, i.e., number of treatments, need to be optimized, IGET is a promising treatment to be transposed to a human clinic.

## Additional files

Additional file 1: **Figure S1.** Partial Irreversible electropermeabilization (pIRE) procedure. (DOCX 285 kb)

Additional file 2: **Figure S2.** Optimization of the delay between Gene-Electro-Transfer procedure and pIRE treatment. (DOCX 145 kb)

Additional file 3: **Figure S3.** Effect of pIRE treatment on tumor volume and survival on transgenic mice. (DOCX 290 kb)

Additional file 4: **Figure S4.** Effect of Hsp70 induction of pIRE treatment (DOCX 427 kb)

Additional file 5: **Table S1.** Biochemistry results of male and female mice of both groups at D15 and D49, mean ± SD. (DOCX 16 kb)

## Data Availability

The datasets analyzed during the current study are available from the corresponding author on request.

## Abbreviations

CD4: Cluster of differentiation 4
CTL: Cytotoxic T lymphocytes
CTLA-4: Cytotoxic T-lymphocyte-associated protein 4
DC: Dendritic cells
EP: Electro-permeabilization
GET: Gene Electro-Transfer
Hsp70: Heat-shock protein 70
HV-MV: High voltage-Medium voltage
IGET: Immune-Gene Electro-Therapy
IL-12: Interleukine-12
INF-γ: Gamma-interferon
NK: Natural killer
PD1: Programmed cell death protein 1
PDL-1: Programmed death-ligand 1
PEF: Pulsed electric field
pIRE: Partial irreversible electroporation
Th1: Helper T cell Type1
