# Supplementary material for: Pre-clinical investigation of the synergy effect of interleukin-12 gene-electro-transfer during partially irreversible electropermeabilization against melanoma
Source: J Immunother Cancer. 2019 Jun 26;7:161. doi: 10.1186/s40425-019-0638-5 (PMC6595571; doi:10.1186/s40425-019-0638-5)
Supplement: Supplementary file 1 — Figure S1. Partial Irreversible electropermeabilization (pIRE) procedure. (DOCX 285 kb) [file 40425_2019_638_MOESM1_ESM.docx]

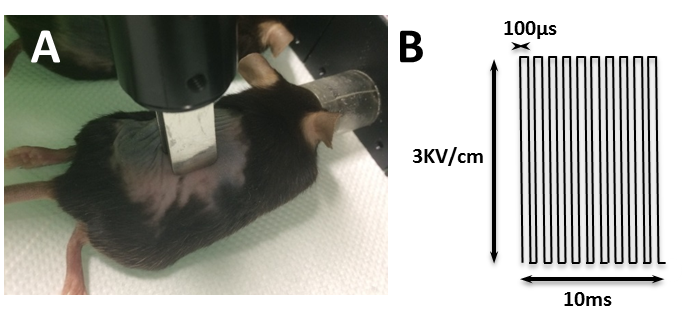


***Supplementary Figure 1: Partial Irreversible electropermeabilization (pIRE) procedure.***

A- The tumor was squeezed between stainless steel, flat, parallel (0.4cm gap) electrodes using Echogel to obtain a good electrical contact. B- Scheme of pIRE pulses: 10 pulses of 1200 V (3 kV/cm), duration 100µs applied with the frequency of 1kHz.
