# Supplementary material for: Pre-clinical investigation of the synergy effect of interleukin-12 gene-electro-transfer during partially irreversible electropermeabilization against melanoma
Source: J Immunother Cancer. 2019 Jun 26;7:161. doi: 10.1186/s40425-019-0638-5 (PMC6595571; doi:10.1186/s40425-019-0638-5)
Supplement: Supplementary file 2 — Figure S2. Optimization of the delay between Gene-Electro-Transfer procedure and pIRE treatment. (DOCX 145 kb) [file 40425_2019_638_MOESM2_ESM.docx]

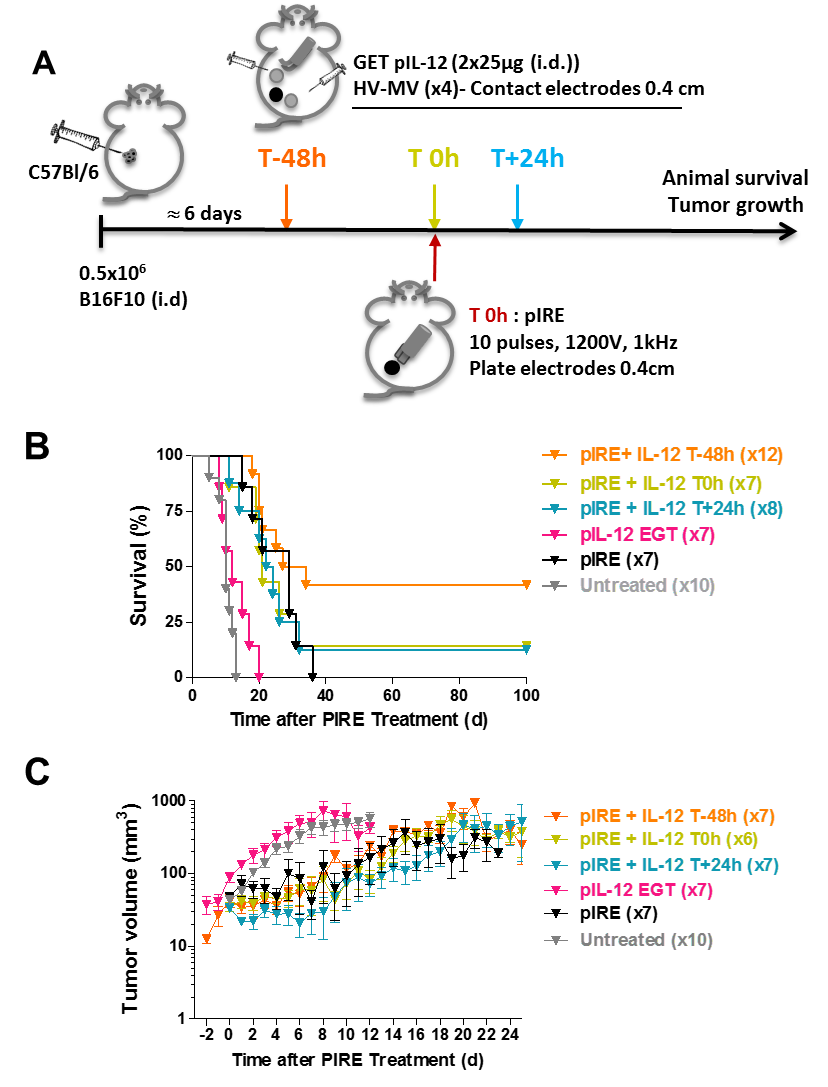


***Supplementary Figure 2: Optimization of the delay between Gene-Electro-Transfer procedure and pIRE treatment.***

B16F10 cells (0.5x106/20µL of PBS) were intradermaly (i.d.) injected in the flank of the mouse (A). Gene electrotransfer (GET) was performed 2 days (T0-48h) before, the day of pIRE treatment (T0), or one day later (T+24h). GET procedure consisted in 2 i.d. injections of 25µg/20µL of plasmid DNA (pIL-12) (grey spots) at the tumor (black spot) vicinity. Stainless steel contact electrodes (0.4cm gap) were used to deliver HV-MV pulses on the skin around the plasmid injection points. HV pulse consisted in one pulse of 100µs duration and 400V amplitude and MV pulse consisted in one pulse of 20ms duration and 100V amplitude. Four repetitions were used at 1Hz frequency.

After treatments, animal survival (B) and tumor growth (C) were evaluated. For tumor growth, complete regression were not presented on the graph. The number of mice are indicated on the graph.
