# Supplementary material for: Pre-clinical investigation of the synergy effect of interleukin-12 gene-electro-transfer during partially irreversible electropermeabilization against melanoma
Source: J Immunother Cancer. 2019 Jun 26;7:161. doi: 10.1186/s40425-019-0638-5 (PMC6595571; doi:10.1186/s40425-019-0638-5)
Supplement: Supplementary file 3 — Figure S3. Effect of pIRE treatment on tumor volume and survival on transgenic mice. (DOCX 290 kb) [file 40425_2019_638_MOESM3_ESM.docx]

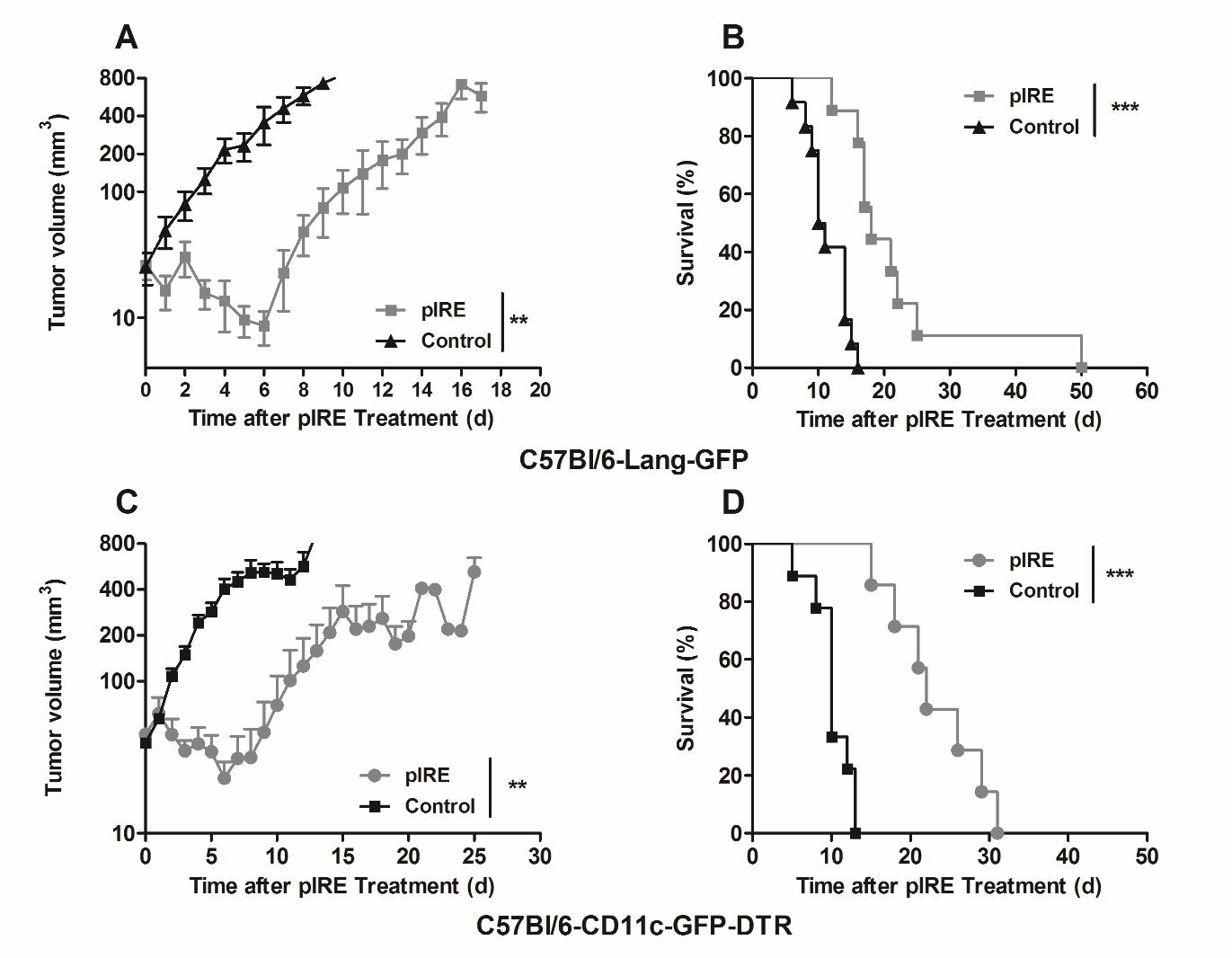


***Supplementary Figure 3: Effect of pIRE treatment on tumor volume and survival on transgenic mice.***

C57Bl/6-Lang-GFP and C57Bl/6-CD11C-GFP-DTR mice were intradermally injected with 0.5x10^6^ B16F10. When the tumor reached a volume of 20 to 30mm^3^, pIRE parameters were applied: 10 square waved pulses of 1200V, duration 100µs, frequency 1kHz. Tumor volume was followed up every day (d) post treatments with a digital caliper. (A) Individual curves of tumor volume for untreated control (▲) and pIRE treated C57Bl/6-Lang-GFP mice (■). Values are means ± s.e.m. **P<0.01, (Mann Whitney test). (B) Survival curves of untreated control (▲) and pIRE treated C57Bl/6-Lang-GFP mice (■). ***P<0.001 (Log-rank (Mantel-Cox) Test). (C) Individual curves of tumor volume for untreated control (■) and pIRE treated C57Bl/6-CD11C-GFP-DTR mice (●). Values are means ± SEM. ***P<0.001, (Mann Whitney test). (D) Survival curves of untreated control (■) and pIRE treated C57Bl/6-CD11C-GFP-DTR mice (●). ***P<0.001 (Log-rank (Mantel-Cox) Test). 9 ≤N≤ 12 tumors per group.
