# Supplementary material for: Pre-clinical investigation of the synergy effect of interleukin-12 gene-electro-transfer during partially irreversible electropermeabilization against melanoma
Source: J Immunother Cancer. 2019 Jun 26;7:161. doi: 10.1186/s40425-019-0638-5 (PMC6595571; doi:10.1186/s40425-019-0638-5)
Supplement: Supplementary file 4 — Figure S4. Effect of Hsp70 induction of pIRE treatment (DOCX 427 kb) [file 40425_2019_638_MOESM4_ESM.docx]

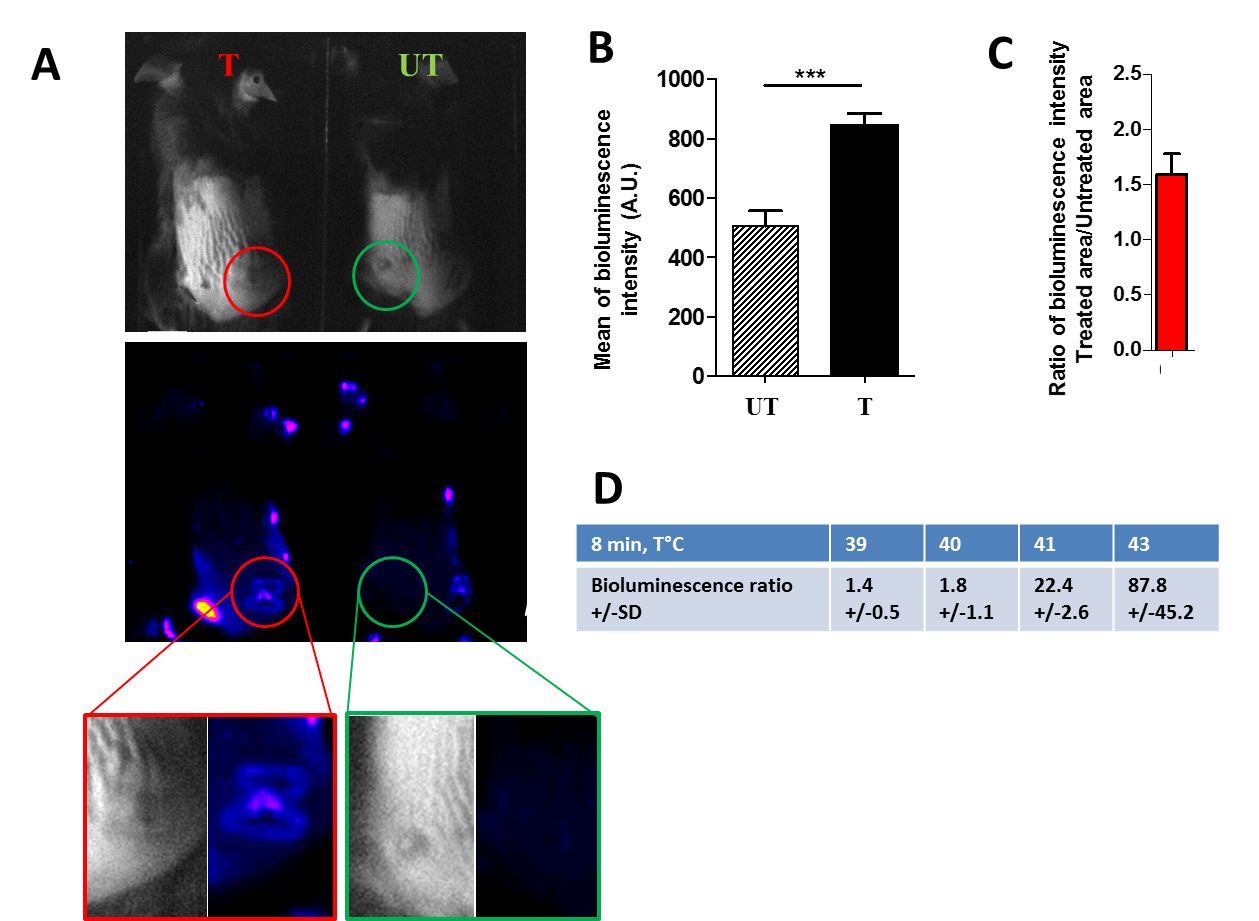


***Supplementary Figure 4: Effect of Hsp70 induction of pIRE treatment***

Transgenic Hspa1b-LucF (+/+) Hspa1b-mPlum (+/+) mice were intradermally injected with 0.5x10^6^ B16F10 cells. When the tumor reached a volume of 20 to 30mm^3^, pIRE parameters were applied with contact electrodes (0.4cm gap): 10 square waved pulses of 1200V, duration 100µs, frequency 1kHz. Bioluminescence imaging of pIRE-induced expression of LucF in these mice was performed 6h after pIRE. Luciferin (3 mg/mouse) was injected intraperitoneally 5 min before imaging (A) Gray images (top) and bioluminescent images (bottom) of transgenic mice, 6h after pIRE treatment (T) or no treatment (UT). High magnifications of treated (left) and untreated (right) tumor areas. (B) Quantification of tumors area mean bioluminescence intensity after pIRE treatment (T) or no treatment (UT). (C) Bioluminescence ratio between pIRE treated (T) or not treated (UT) tumors area. (D) Bioluminescence ratios determined after thermal stress between the leg exposed to the indicated temperature (°C) water-bath for 8 min and the not exposed leg. Values are means ± s.e.m., n=3, ***P<0.001, (Two-way ANOVA analysis).
