# Supplementary material for: Pre-clinical investigation of the synergy effect of interleukin-12 gene-electro-transfer during partially irreversible electropermeabilization against melanoma
Source: J Immunother Cancer. 2019 Jun 26;7:161. doi: 10.1186/s40425-019-0638-5 (PMC6595571; doi:10.1186/s40425-019-0638-5)
Supplement: Supplementary file 5 — Table S1. Biochemistry results of male and female mice of both groups at D15 and D49, mean ± SD. (DOCX 16 kb) [file 40425_2019_638_MOESM5_ESM.docx]

***Supplementary* Table 1:** **Biochemistry results of male and female mice of both groups at D15 and D49, mean ± SD.** 2x25µg of pIL-12 were injected intradermally in the healthy skin of female and male C57Bl6 mice. Electrotransfection was realized using HV-MV electrical parameters. Biochemistry parameters (Glucose, Urea, Creatinin, Triglycerides, Cholesterol, Protein, Albumin, GOT, GPT, Calcium, Phosphore, Chloride, Sodium, Potassium, Bilirubin) were determined on 2 females and 2 males mice treated with control and 4 females and 3 males mice treated with GET sacrificed at D15 and on 2 females and 2 males mice treated with control and 4 females and 4 males mice treated with GET sacrificed at D49.

| Male mice |  | **D15** | | **D49** | |
| --- | --- | --- | --- | --- | --- |
| **Parameters** | **Unity** | **Group 1** | **Group 2** | **Group 1** | **Group 2** |
|  |  | **Control** | **EGT** | **control** | **EGT** |
| **Glucose** | g/l | 2,49 ± 0,52 | 2,57 ± 0,28 | 1,44 ± 0,23 | 2,06 ± 0,19 |
| **Urée** | g/l | 0,38 ± 0,02 | 0,48 ± 0,06 | 0,46 ± 0,02 | 0,49 ± 0,08 |
| **Créatinine** | mg/l | <2 | <2 | <2 | <2 |
| **Triglycérides** | g/l | 0,79 ± 0,31 | 1,33 ± 0,25 | 1,43 ± 0,05 | 1,26 ± 0,58 |
| **Cholestérol** | g/l | 0,77 ± 0,08 | 1 ± 0,03 | 0,93 ± 0,11 | 1 ± 0,11 |
| **Protéines** | g/l | 45,5 ± 2,4 | 44,9 ± 2,59 | 44,15 ± 0,78 | 48,85 ± 11,38 |
| **Albumine** | g/l | 27,4 ± 0,99 | 26,33 ± 0,81 | 26,9 ± 0,14 | 27,05 ± 1,16 |
| **GOT** | U/l | 74,5 ± 44,55 | 185 ± 143 | 102,5 ± 17,68 | 101,75 ± 43,11 |
| **GPT** | U/l | 32,5 ± 3,54 | 150 ± 116,48 | 69,5 ± 6,36 | 58,25 ± 24,92 |
| **Calcium** | mg/l | 92,35 ± 1,34 | 89,73 ± 3,59 | 92,35 ± 5,73 | 94,75 ± 4,53 |
| **Phosphore** | mg/l | 72,7 ± 3,96 | 76,1 ± 6,68 | 67,25 ± 10,54 | 66,63 ± 6,91 |
| **Chlorures** | mEq/l | 102,65 ± 1,63 | 96,57 ± 0,6 | 102,7 ± 1,27 | 103,33 ± 0,88 |
| **Sodium** | mmol/l | 151,54 ± 1,99 | 162,96 ± 1,54 | 192,62 ± 3,15 | 195,41 ± 0,68 |
| **Potassium** | mmol/l | 3,84 ± 0,2 | 4,73 ± 0,6 | 3,77 ± 0,66 | 4,05 ± 0,35 |
| **Bilirubine** | mg/l | 2,7 ± 1,27 | 1,57 ± 0,4 | 0,75 ± 0,07 | 1,15 ± 0,06 |
| **Albumine/globuline ratio** | mmol/l | 1,52 ± 0,06 | 1,42 ± 0,09 | 1,56 ± 0,05 | 1,39 ± 0,42 |
| Female mice |  | **D15** | | **D49** | |
| **Parameters** | **Unity** | **Group 1** | **Group 2** | **Group 1** | **Group 2** |
|  |  | **Control** | **EGT** | **Control** | **EGT** |
| **Glucose** | g/l | 1.99 ± 0.53 | 1.67 ± 0.42 | 2.79 ± 0.88 | 2.17 ± 0.43 |
| **Urea** | g/l | 0.52 ± 0.02 | 0.51 ± 0.06 | 0.34 ± 0.03 | 0.47 ± 0.02 |
| **Creatinin** | mg/l | <2 | <2 | <2 | <2 |
| **Triglycerides** | g/l | 1.43 ± 0.28 | 1.14 ± 0.27 | 0.69 ± 0.04 | 1.11 ± 0.42 |
| **Cholesterol** | g/l | 0.79 ± 0.07 | 0.85 ± 0.03 | 0.83 ± 0.07 | 0.78 ± 0.07 |
| **Protein** | g/l | *50.00 ± 1.56* | *46.83 ± 1.91* | 46.45 ± 0.92 | 44.63 ± 1.88 |
| **Albumin** | g/l | *30.60 ± 0.99* | *29.13 ± 1.43* | 29.65 ± 0.78 | 28.95 ± 0.75 |
| **GOT** | U/l | *234.0 (n=1)* | *100.5 ± 39.6* | 120.5 ± 50.2 | 140.5 ± 71.9 |
| **GPT** | U/l | *80.0 ± 21.2* | *59.0 ± 36.1* | 26.0 ± 14.1 | 69.0 ± 27.9 |
| **Calcium** | mg/l | *95.3 ± 0.2* | *91.6 ± 5.7* | 95.4 ± 0.7 | 98.1 ± 1.8 |
| **Phosphore** | mg/l | 86.9 ± 19.0 | 79.3 ± 6.0 | 72.7 ± 1.6 | 77.5 ± 5.5 |
| **Chloride** | mEq/l | 103.1 ± 0.4 | 102.9 ± 1.3 | 101.4 ± 0.3 | 103.5 ± 2.2 |
| **Sodium** | mmol/l | 153.1 ± 6.0 | 156.7 ± 1.6 | 184.4 ± 4.8 | 187.2 ± 2.3 |
| **Potassium** | mmol/l | *3.08 ± 2.52* | *4.12 ± 0.51* | 3.75 ± 1.07 | 3.84 ± 0.47 |
| **Bilirubin** | mg/l | *2.10 (n=1)* | *2.25 ± 0.54* | 1.45 ± 0.07 | 1.38 ± 0.13 |
| **Albumin/globuline ratio** | mmol/l | 1.58 ± 0.01 | 1.64 ± 0.04 | 1.76 ± 0.03 | 1.85 ± 0.11 |
